# Providers’ insight into quality mental health services – Context-Mechanism-Outcome (CMO) approach

**DOI:** 10.1186/s12913-025-12372-x

**Published:** 2025-02-17

**Authors:** Eric Badu, Anthony Paul O’Brien, Rebecca Mitchell, Akwasi Osei

**Affiliations:** 1 Social Policy Research Centre, The University of New South Wales, Sydney, NSW Australia; 2 School Nursing and Midwifery, Monash University, Clayton, VIC Australia; 3 Macquarie Business School, Macquarie University, Macquarie Park, Ryde, NSW Australia; 4 Ghana Mental Health Authority, Ghana Health Services, Accra, Ghana

**Keywords:** Quality improvements, Mental health services, Realistic evaluation, Context, Mechanism, Outcome, Ghana

## Abstract

**Background:**

Evaluation frameworks are relevant to understanding health service providers’ views regarding existing services and possible improvements, but their application to mental health services is limited, particularly in Low Middle-Income Countries.

**Aim/question:**

To identify a program theory that explains the contextual factors and mechanisms that could enhance mental health service outcomes in Ghana.

**Method:**

A three-phase approach was followed: initial theory and assumption, analysis, and CMO configuration. Systematic reviews were used to develop a middle-range theory and assumptions in phase 1. A purposive sample of 30 mental health professionals was recruited to participate in in-depth interviews in phase 2. Thematic analysis was used to analyze the qualitative data and further configure the CMO in phase 3.

**Results:**

The analysis identified five CMO configurations: ripple effects and financing source sustainability; unavailability of modern equipment and logistics to support holistic services; promoting inclusivity and geographical proximity of services; information, sensitization, and awareness encourage mental health quality; and monitoring and evaluation improve mental health service quality.

**Conclusion:**

This study concludes that government stakeholders should integrate mental health services into the ongoing insurance policy and provide adequate modern equipment and logistics. Moreover, mechanisms and priorities given to vulnerable consumers should be integrated into policies.

**Supplementary Information:**

The online version contains supplementary material available at 10.1186/s12913-025-12372-x.

## Background

Over the past years, stakeholders and researchers have been advocating for innovative approaches to measure the outcomes of programs and interventions. Several theoretical and conceptual frameworks have been developed and implemented to measure service outcomes [1–4]. For example, several middle-range theories (Donabedian, Penchansky and Thomas) are used to measure mental health outcomes from the perspective of the public, mental health professionals (MHPs), consumers, and family caregivers. These conventional or middle-range theories are mostly applied using structure, process, and outcome indicators [1, 4]. Structure indicators typically comprise the health system indicators, including the system availability, accessibility, adequacy, and affordability, as well as consumers’ awareness and individual factors [4]. Some researchers have categorized these frameworks into organizational, individual, and environmental factors that contribute to the quality of mental health services. Next, several proponents of middle-range theories have described the process for focusing on providers’ technical competency and their interactions with their consumers who receive services. Lastly, the outcome, which is the most relevant measure of quality, mostly considers the objective and subjective changes in the lives of mental health service consumers after treatment.

Several studies highlight the significant contributions of these middle-range theories, for example, in informing quality assurance management, supporting ongoing monitoring, and improving mental health services [1, 4]. These quality improvements have widely been understood from the direct clinical outcomes, mental health system reports, and consumer symptomology. However, advocates of mental health quality assessments have recommended that it is necessary to refine quality measures, invest in information technology, and foster a culture of measurement-based care that could enhance the outcomes of mental health services [2, 3, 5]. Specifically, such arguments have identified the need to incorporate the views of consumers and family caregivers toward ensuring quality improvements. In particular, such proponents are widely perceived as helping to promote recovery-oriented services, thus emphasizing the partnership between MHPs and consumers [2, 4–6]. Understanding mental health quality from the consumers’ perspective not only promotes partnership but improves their knowledge about the treatment as well as treatment functionality (self-care, getting along with people, participation [2, 4].

As the debate progress, evaluation experts have argued that a problem-solving approach should be used to integrate these differences, which would thus value all stakeholders but clarify aspects that work and do not work [7]. Given this view, evaluation frameworks have recently been proposed to measure health service outcomes [7], which could be applied to mental health services. For example, evaluation methods, such as formative and summative, as well as development [8] are increasingly applied in real services and interventions. Other evaluators and researchers have recently applied realistic evaluation as well as ripple effects mapping [9] as promising evaluation methods to construct and reconstruct program theory, which includes logic modelling, logical frameworks, outcome hierarchies, and antecedent target measurement [7, 10]. These evaluation frameworks are used to simplify a complex process (providing structure and guidance for the evaluation processes), promote meaningful evaluation, and thus, identify and include stakeholders, explain reasons for outcomes, generate transferable lessons, and identify mechanisms driving or inhibiting change [7].

Although these frameworks are relevant to understanding the views of service implementers—for example, MHPs—regarding how services work and could be improved to enhance the social and psychological well-being of consumers, limited studies have applied such frameworks to the mental health field. The existing frameworks have been applied widely to maternal health and other health services (Abimbola et al., 2016). In particular, most of the frameworks evaluating services, such as mental health, are typically applied to those of developed countries, including the United States, the United Kingdom(Bertotti, Frostick, Hutt, Sohanpal, & Carnes, 2018; Greenhalgh et al., 2009), Sweden, and Germany, with practically limited application to understand mental health services in developing countries.

Although there are increasing research efforts to improve mental health quality assessments in Ghana, none has attempted to use innovative approaches, such as realist evaluation. Most studies on the country’s mental health services are limited to examining the policy process and implementation as well as service strengths and weaknesses, using conventional middle-range theories. Given this gap in research problem-solving, we employ a realistic evaluation methodology to (a) identify the program theory for understanding the quality of mental health services and (b) explore the contextual factors and mechanisms that could achieve the desired outcomes from mental health services in Ghana.

## Methods

### Research design and approach

We used the interpretive case study research approach for this study. Specifically, the approach used to implement this case study is a realistic evaluation. A realistic evaluation provides a unique perspective on programs and services grounded in theory or practice [11, 12]. This evaluation offers ways to identify whether, when, why, and for whom programs or services work and the circumstances under which they work [13, 14]. The realistic evaluation assumes that innovations, programs, and services will work only in particular circumstances and that the central formulation is to identify the mechanisms that work and the contexts in which they produce outcomes [15]. This component is explained by Pawson and Tilley [16] through the formula: Context + Mechanism = Outcome (Table 1). We chose the realistic evaluation approach owing to its sensitivity to context and methodological flexibility. This allowed us to simultaneously explore the design and provision of mental health services in a manner congruent with theory. The central tenet of the realist methodology is that a service may work differently in different contexts [17, 18].

**Table 1:** Operationalization of concepts

Concepts	Definitions
Middle-range theory (MRT)/Initial Program Theory	Middle-range theory” are those that can be tested with the observable data and is not abstract to the point of addressing larger social or cultural forces [17]
Quality mental health services	Quality mental health services under this realistic evaluation were operationalized as evidence-based services that conform to best practice to achieve desired outcomes
Context	Context refers to salient conditions that are likely to interact, influence, modify, facilitate or hinder the quality of mental health services and its effectiveness. In our realistic evaluation, it may include the policy background, cultural, historical, and mental health systems as well as organizational setting where the services are being delivered
Mechanisms	Mechanisms describe what makes services work. They are not the observable machinery of service activities, but the response that interaction with a service activity triggers (or does not trigger) in the lives of consumers
Outcome	Outcomes describe the intended and unintended consequences of the change efforts by mental health services. In our study, the outcome includes the clinical and non-clinical outcomes, including the quality of life, recovery, symptoms, functionality, physical health, psychological wellbeing, and satisfaction. The outcome also includes MHPs experiences identified through the in-depth interviews
CMO Hypothesis	Context + Mechanisms = Outcome. The CMO hypotheses underlying this realistic evaluation was that Contextual factors together with Mechanisms could help achieve an Outcome of mental health services

Mental health quality assessments have traditionally been tested and evaluated through middle-range theories (e.g., Donabedian theory) to explore the structure, process, and outcomes, but such a model has never been evaluated from a realist perspective. This theory-based evaluation involves developing and refining a program theory, which includes contexts, mechanisms, and outcomes. This method reveals how mechanisms and outcomes are constrained by individual, program-specific, and societal contexts or health systems. Thus, the focus is not just on whether or not the mental health service “works for consumers” but also on how, for whom, and in what contexts it “works.” Realist evaluation addresses these questions by developing the context–mechanism–outcome (CMO) configuration [19].

This approach was relevant because of its ability to provide insights through detailed contextual analysis. We selected three psychiatric hospitals in Ghana as cases. We used the mental health services provided in each psychiatric facility as a case but in the context of a shared program theory. In the realistic evaluation, qualitative data was collected through semi-structured interviews, and a review of the literature and program information. Articles on the literature review [20, 21] the conceptual framework [4, 22] and quantitative components [23] have been published. The qualitative data helped to explore the subjective experiential perspective and are typically based on data saturation. This article focuses on the qualitative component, which draws on the principles of realist evaluation to evaluate MHPs’ realistic perspectives underpinning the context and mechanism toward achieving quality mental health services.

#### Phase 1: Initial theory and assumption

We conceptualized the realistic evaluation cycle into phases, commencing from the initial theory development phase [12, 18]. In this evaluation, we relied on Donabedian [4] and Thomas and Pachensky’s theories as middle-range theories to describe the explanatory pathways related to the health system structure and the process factors associated with mental health service quality. Donabedian, and Thomas and Pachensky, explain the structure of health systems and the process to measure the quality of mental health services. We iteratively refined these working theories as an initial program theory (IPT) during the evaluation [19]. Then, we empirically tested the evaluation data (e.g., qualitative data) to develop a final program theory that explains how and in which conditions the services work (Table 3). Specifically, we used the IPT theory to identify mechanisms that hindered or facilitated mental health service quality based on MHPs’ perspectives. Then, we used their experiences based on their interactions with the services and consumers to refine the program theory. The objective was to better understand why and when innovations and different approaches work for consumers [19].

**Fig. 1:**
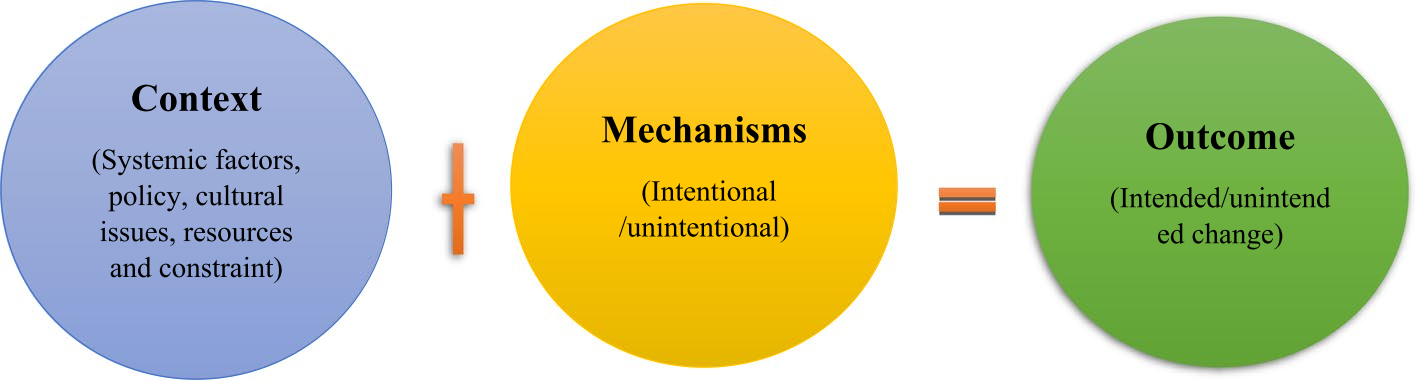
Initial program theory

The next step of the realist evaluation process involved developing the underlying assumptions to articulate the program theory [11, 12, 24]. The CMO hypotheses were derived from the IPT (Fig. 1). Given that the realistic evaluation uses flexible methods and approaches, the study was based on real services and people. The mental health services involved are complex and holistic, and thus, the study involved layers of reality—and these layers of interest are real, if not always tangible. For example, the realistic evaluation aimed to understand the links between the components of the layers of context, mechanism, and outcome and was less interested in a descriptive account of practitioners’ feelings and perceptions.

#### Phase 2: Fieldwork (recruitment and data collection)

As part of program theory articulation, the research team used qualitative approaches, such as in-depth interviews and field notes to collect data from MHPs. The fieldwork was performed in three psychiatric facilities to provide greater contextual information in preparation for CMO articulation. The choice of psychiatric facilities and psychiatric units is based on their geographic location and helped in obtaining a representative sample of participants nationwide. The field notes was used to capture information on the working environment, the dynamics of care, and the consumer flow and treatments during a six-month period. We organized an interactive discussion and formative feedback with the clinical coordinators in each of the psychiatric facilities. The clinical coordinators facilitated the recruitment of MHPs (psychiatrists, mental health nurses, occupational therapists, clinical psychologists, social workers, and art therapists). For example, we purposively recruited MHPs who have worked for at least three years in the respective psychiatric facility and provided daily routine mental health care. In collaboration with the clinical coordinators, we reviewed the list of MHPs in each of the selected facilities and selected those who met the inclusion criteria.

The selected MHPs were invited to participate in the study via emails, postal mails, or phone calls. The invitation contained a letter of introduction, participant information sheets, and consent forms. The letter explained the purpose of the research, its inclusion criteria, and the recruitment process. The phone calls were only used to confirm the prospective participants name and professional identify. The researchers then followed up by sending an email invitation and or delivered the recruitment materials to the participant in-person. The head of each psychiatric facility sent a memo detailing the research and advertised it on the notice boards in each department. A total of 38 MHPs were invited to participate in the study. Eight declined the invitation, leaving a total sample of 30 MHP participants. Those who declined to participate were psychiatrists, clinical psychologists and mental health nurses. Given the methodological flexibility of realistic evaluation, we recruited MHPs until data saturation, that is, until no new information was forthcoming from subsequent interviews.

Phase 2 of the realistic evaluation conformed to the ethical guidelines of the Helsinki declaration. For example, the Human Research Ethics Committee, University of Newcastle (Approval No.: H-2019–0082) as well as the Ethical Review Committee of the Ghana Health Services (Approval No: GHS-ERC 003/07/19) approved the study. The in-depth interviews captured information regarding the CMOs and were conducted using a structured interview guide (See Appendix 1). Relevant health systems, or contextual factors, according to the middle-range theory (or IPT) were used to develop the interview guide [4]. A demographic characteristics questionnaire was used to collect information on participants’ age, gender, educational qualifications, marital status, primary occupation, profession, and years of experience.

As part of the in-depth interviews, all the MHPs were briefed about the research objectives, procedures, and the consent process. The interviewer read the questions on the interview guide (additional file) to the participants and recorded (with permission) their responses, using an audio-tape recorder. All the in-depth interviews were conducted in the English language, which is the primary language of conversation in formal educational settings in Ghana. The in-depth interviews were conducted at the psychiatric facilities, that is, at the clinical staff common rooms, separately; no interview was witnessed by a clinical coordinator or any service provider.

#### Phase 3: Analysis and configuration of CMO

The CMO configurations informing the analysis were developed, confirmed, and discussed by the research team before being applied for the analysis. The analysis and CMO configurations followed several steps. First, the field notes information, as well as in-depth interviews, were analyzed using thematic analysis. The thematic analysis involved identifying codes and patterns emerging from the interviews and was conducted in line with the approach of Braun and Clarke [25] to categorise and connect strategies (e.g., by transcribing, reading, and becoming familiar with the data; generating initial codes; searching for themes; reviewing themes, and rigorously interpreting data) for developing a realist theory.

A total of 30 de-identified audio recordings were transcribed into a word document by an independent transcriber and then checked by the interviewer. Then, the transcribed data were entered into NVivo 12 for analysis. A standardized codebook defining domain-specific codes was developed (Table 3). In all, 73 inductive codes and 588 references were initially developed from the transcribed data. The codes were assigned to each relevant piece of interview data based on the CMO notation or themes. We organized the analysis narratively according to the specific CMO domain. Memos were written throughout the coding process to record emerging conceptual links and observations from the data. As illustrated in Table 3, the patterns, or links of CMO configuration across cases were proposed. The patterns denote the causal pathways, and thus, the contextual elements that could trigger, or influence mechanisms, to produce a mental health service outcome. The analysis identified five CMOc across the interviews (Table 3). The findings have been arranged according to these CMO configurations. The most prominent quotations and words from MHPs that were relevant to each of the CMOs are provided.

## Results

### Background information

As illustrated in Table 2, the average age of MHPs was 36.4 years. Most of the MHPs (17/30; 56.67%) were females, whilst 43.33 were males. The majority of MHPs (19/30; 63.33%) were married, 33.33% were singles. More than a third (46.66%) of the MHPs were Registered Mental Health Nurses, whilst 23.33% were Psychiatrist. The highest level of education among the MHPs were at the Postgraduate degree (eg. MPH, MSc, MPhil and MBChB). The average years of working experience of the MHPs was 8.4 years.

**Table 2:** Background information of MHPs

*Variable*	*Frequency*	*Percentage*
*Gender*		
Male	13	43.33
Female	17	56.66
Marital status		
Married	19	63.33
Single	10	33.33
Separated	1	3.33
Qualification		
Masters degree	7	23.33
Bachelor of Medicine, Bachelor of Surgery (MBChB)	7	23.33
Bachelor’s degree	12	40.00
Diploma	4	13.33
Primary occupation		
Psychiatrist	7	23.33
Psychologist	4	13.33
Occupational therapist	3	10.00
Mental health nurse	14	46.66
Art therapist	1	3.33
Social worker	1	3.33
Age*		
21 – 30	8	26.66
31 – 40	16	53.33
41 – 50	3	10.00
51 – 60	3	10.00
Years of working experience +		
3 – 8	15	50.00
9 – 14	12	40.00
15 – 20	2	6.66
21 – 25	1	3.33

*Min/Max (Mean); 26/56 (36) +Min/Max (Mean); 3/24 (8.4)

### CMO configurations from the analysis

As illustrated in Tables 3, five CMOc were identified by analyzing the interviews and field notes. The findings have been arranged according to these CMOcs and further supported with the relevant verbatim quotations from the MHPs.

**Table 3:** CMOcs from the configuration

CMOcs from the configuration	Context + Mechanism = Outcome
*CMOc configuration 1a: Ripple effects and sustainability of financing sources*	The government was identified as a mandatory source of financing mental health services. Mental health services were also over-reliant on internally generated funds and external sources (e.g., NGOs, DFID, UK-AID, churches and individual givers [**Context**]) The mandatory source allocated a budget for running mental health institutions and paying salaries to the mental health workforce. The process of financing services was not adequately implemented, leading to out-of-pocket payments, the lack of insurance coverage/NHIS not covering mental health services, and the high cost of services (**Mechanisms**). The unintended negative ripple effects influence access to services, particularly continuity and clinical review, makes services unaffordable, and reduces clinical review. The ripple effects of financing sources increase sustainability issues (**Outcome**)
*CMOc configuration 1b: Ripple effects and medication financing capability*	The government was identified as the mandatory source of financing psychiatric medications. Mental health services were also over-reliant on internally generated funds and external sources (e.g., NGOs, DFID, UK-AID, churches and individual givers [**Context**]) The mandatory sources of medications had an infrequent supply, leading to high costs for quality medication, purchases of medication from outside the psychiatric pharmacy on a cash-and-carry basis, the high cost of purchasing psychiatric medication, and limited local protocols to inform medication prescription (**Mechanisms**). The unintended negative ripple effects were that consumers were unable to afford the cost; clinical reviews reduced; consumers’ ability to secure/purchase quality medications was adversely affected; they purchased alternate lower-cost drugs, reduced the medication quantity, or went home without medication; noncompliance as well as adverse side effects increased; and the quality of services was poor, which increases adverse side effect or may cause the death of consumers (**Outcome**)
*CMOc configuration 2: Unavailability of modern equipment and logistics to support holistic services*	Psychiatric facilities operated a holistic/integrated philosophy of care (e.g., medication management, intensive community treatment services, forensic services, rehabilitation services, psychological services, admissions, psychotherapy, rehabilitation hospitalization, daycare, home help, outpatient visits as well as clinical recovery, psychosocial and psychoeducation, promoting activities in occupational therapy, and promoting recovery services [**Context**]) However, MHPs lacked the relevant equipment and logistics (e.g., their respective facilities had malfunctioning electroconvulsive therapy machines and did not have a straitjacket and equipment for comprehensive psychological and vocational services) to perform their work (**Mechanism**),which could compromise the quality of mental health services, increase average working hours spent on consumers, cause inconvenience, slow down clinical recovery, delay the recovery and management of postpartum psychosis, lead to the occurrence of injuries among service providers and consumers, and endanger the privacy and confidentiality of consumers (**Outcome**)
*CMOc configuration 3: Promoting inclusivity and geographical proximity of services*	Psychiatric facilities aspire to make services accessible to all consumers (**Context**) by promoting inclusive mental health services for all consumer groups through scheduling services for clinical days or contacts, prioritizing services for elderly consumers and people with disabilities (e.g., allowing them to skip queues and providing support to meet their needs), disaggregating in-patient wards by category of consumers and services, and paying for the costs incurred by consumers who are vagrant or with chronic disease and have no finance or family caregivers (**Mechanism**) to promote quality mental health services; increase clinical contact, equity and inclusive services; enable consumers to access a wide range of specialist services; improve contact care; reduce the waiting time at the psychiatric facilities; and promote inclusive mental health services (**Outcome**). Moreover, accessible psychiatric facilities by MHPs (**Context**), promote outreach services with support from organizations (**Mechanism**) to reduce geographical proximity issues (**Outcome**)
*CMO configuration 4: Information, sensitization and awareness encourages mental health quality*	Several platforms, such as psycho OPD sessions, PR office as well as media (radio and TV; social media, such as Facebook, Instagram, and Twitter), and outreach programs (**Context**), are used to create awareness (e.g., in communities, schools, churches, and the public), provide psychoeducation about medication and side effects, provide an educational session or workshop to enlighten MHPs on new medications and collaboration with stakeholders (churches, special educators, physiotherapists, social welfare, faith-based healers, police, NGOs and schools [**Mechanism**]), to improve the public knowledge about services, inform the public about the available mental health treatments, reduce stigmatizing attitudes, improve the quality of the services, manage potential side effects, promote evidence-based treatment, enlighten providers on new medications, and promote the quality of, and access to, mental health services (**Outcome**)
*CMOc configuration 5: Monitoring and evaluating improve mental health service quality*	The quality assurance team conducts quality assessments through internal and external clinical peer reviews, as well as monthly, quarterly, and annual reports and meetings (**Context**), using parameters, such as whether MHPs follow the mandated dress code and communicate clearly and politely with consumers; whether consulting rooms meet the basic requirements; and whether medications are prescribed correctly. The team uses therapeutic interactions and assessment sheets, or verbally asks the consumers regarding their activities or observes them (**Mechanism**) This helps to monitor and evaluate the progress of the mental health services (e.g., identify the strengths and weakness in the services), improve the quality of mental health services, set goals and targets, and identify challenges and ways to improve the services (**Outcome**)

### CMO configuration 1a: Ripple effects and financing source sustainability

This CMO describes the ripple effects of the financing sources of services, and their sustainability in providing financial risk protection. Most MHPs narrated that the government was mandated as the financing source of mental health services, together with internally generated funds and external sources (e.g., international organizations, such as non-governmental organizations [NGOs] and UK AID, funded by the UK Government’s Department for International Development [DFID; churches; and individual givers). Further, most of them stated that the government allocated a budget for running mental health institutions and for paying salaries to the mental health workforce and also noted that the provision of financing services by the mandated source was inadequate. For example, they mentioned the lack of insurance coverage, which leads to out-of-pocket payments (e.g., personal payments or by family caregivers), and the high cost of services. Some MHPs mentioned that consumers finance the cost associated with consultation, admission, medication, occupational therapy, and psychological services. The MHPs mentioned that the existing National Health Insurance Scheme (NHIS) does not cover mental health services, as exemplified in the following quotation:

That is our problem because NHIS doesn’t cover the drugs; at the time when the NHIS was being initiated, the drugs were free, so they didn’t consider to add our diagnosis to the scheme; so up to now, the drugs are not being covered by NHIS. So when patients are admitted to the facility for about a month, they will have to pay for everything, including food, and we also prescribe their drugs for them … so it is a huge problem for us, so for us, we are pleading that the Mental Health Authority should intervene so that the drugs will be covered under the scheme so that the cost of drugs and feeding will reduce for the patients. (Participant 1; Registered Mental Health Nurse; Facility 3)

Most MHPs narrated that the out-of-pocket payments/high costs of mental health services have a ripple effect on the mental health services, particularly on the continuity in accessing services as well as on clinical reviews. Some MHPs felt that recognizing that people with mental illness are at higher risk of unemployment and poverty could suggest that they are unable to afford the treatment cost. Sometimes, consumers may feel the need to visit the facility for clinical review; however, the cost impedes them from accessing such services, as elaborated by an occupational therapist:

It affects access because if I want to access the best mental health service, it means I (consumer) have to pay, and you know most of our people they go home and they are unemployed; most of their families are not so supportive and so, obviously, if the condition arises, they may not come to the hospital to even access the service because they know when they come here they have to pay a lot of money. They genuinely can't afford, and I know there are a lot of statistics that have gone around to say that people who have disabilities and mental illness are mostly poor, so I mean they paying for services are going to affect access to the services; not everyone would be able to come and access the services; that is how come they go to the faith healers. (Participant 11, Facility 2)

### CMO configuration 1b: Ripple effects and medication financing capability

This case highlights the ripple effects and the sustainability of financing sources in relation to the purchasing of psychotherapeutic medication. The majority of MHPs narrated that the government was identified as the mandatory source (i.e., central medical stores through the Ghana Health Services) of financing psychotherapeutic medications. However, the provision of financing services by this source was inadequate, leading to infrequent supply of medications, limited protocols to inform medication prescription, and over-reliance on external support and consumers. Further, most of them expressed that the inadequate source of financing medications leads to several challenges, including the high cost of purchasing psychotherapeutic medications and the purchase of medications from pharmacies other than the central medical stores or from private pharmacies. Some MHPs noted that the prescription of medications by a pharmacist other than a hospital pharmacist resulted in increasingly adverse side effects for consumers and numerous complaints of poor-quality medications being supplied by private pharmacies. Moreover, the reliance on non-hospital sources resulted in miscommunication regarding medication as well as the unavailability of specific medications from such sources. The following participant MHPs described this process:

Sometimes, they complain that buying it from outside is expensive, and sometimes, most of the pharmacies they go to don’t stock such psychiatric medication; so sometimes, we do have challenges of medications running out, we don’t almost always have theirs; sometimes, they do run out. (Participant 17; Registered Mental Health Nurse; Facility 2)

These things happen because they buy the drugs from outside the hospital, but if it was the hospital that was supplying the drugs, they will not buy cheap drugs but quality ones and those too are not available; so if the patient comes and we do not get some from our pharmacy shop, we will have to let them go to Bantama pharmacy shops, and the drugs there are China made and cheap. (Participant 1; Registered Mental Health Nurse; Facility 3)

Big challenge is that since they are buying the medications from outside, a lot of them as pharmaco-vigilance, some of them are not of good quality; so when the patients take those medications, they get adverse drug reactions that sometimes lead to death… because there are some patients when you prescribe drugs for them and they take it to the pharmacy, they are given different medications. (Participant 3; Registered Mental Health Nurse; Facility 3)

Further, some MHPs noted that when consumers are unable to bear the higher cost of psychiatric medications, they resort to purchasing alternate lower-cost drugs, reducing the medication quantity, or going home without the medications. These practices sometimes result in increasing non-adherence as well as adverse side effects, as echoed by two clinical psychologist participants:

Yes, that is what it is, and that also affects medication compliance or treatment compliance because when the person does not come and defaults for a while and you find out, they will tell you they don’t have money to come because when they come they have to spend money, and so without money they do not turn up, and that is a big problem. (Participant 18, Facility 2)

If people get here and they get discouraged because of the prices that they have to incur, people come and they see the doctors, and then they have prescribed medication, and they go to the pharmacy, and then they go back home because they think that they cannot purchase the medication. (Participant 4, Facility 3)

### CMO configuration 2: Unavailability of modern equipment and logistics to support holistic services

The majority of MHPs noted that the psychiatric facilities are expected to operate a holistic/integrated philosophy of care (e.g., medication management, intensive community treatment services, forensic services, rehabilitation services, psychological services, admissions, psychotherapy, rehabilitation hospitalization, daycare, home help, outpatient visits, clinical recovery, psychosocial and psychoeducation services, promoting activities in occupational therapy, and promoting recovery services). However, some psychiatric facilities were confronting challenges in adhering to this philosophy of care. For example, certain facilities had limited intensive community treatment services, forensic services, or transitional support, which included recovery-oriented services (e.g., personal recovery-focused and evidence-based therapeutic group programs). Most MHPs also commented that although they have been trained on the theories and evidence-based treatment, they lacked the relevant modern equipment and logistics to deliver quality mental health services. For example, the MHPs explained that equipment, such as a straitjacket or an electroconvulsive therapy (ECT) machine, was either unavailable, malfunctioning, or old:

We used to have our electroconvulsive therapy (ECT), but the machine has broken down, so we do not use it or provide that service anymore, and that is our problem there and we sometimes refer them to the general medicine for ECT; some also need scans and we refer them to the general side, but we do not have that equipment or machines here. (Participant 1; Psychiatrist; Facility 3)

Further, some MHPs narrated that adequate equipment and logistics could enhance the service delivery, but the lack of such equipment seriously compromises the quality of mental health services. They highlighted that this lack increases the average working hours they spend on consumers, causes inconvenience, slows down the clinical recovery of patients, and leads to the risk of injury during restraining. In particular, Participant 6 and Participant 26, who have four and five years of experience, respectively, shared that although ECT is useful in managing postpartum psychosis, the lack of such equipment usually delays the recovery from, and the management of, postpartum psychosis:

It affects the quality; so like now, for example, this one that they just called me about, a woman who has postpartum psychosis, the first-line treatment should have been electroconvulsive therapy and that is what we would have done in the past, but it is not available; so now, we have to use medication, which means that it will take a longer time to treat the psychosis, which means that it will affect her ability to care for her baby and things like that; it has ripple effects, so apart from that, there are many other conditions that we need an ECT machine to treat, urgent or emergent conditions, schizophrenia and things like that, but it is not available; so you have to go the old-fashioned long route and that affects the length of stay, quality in so many ways, cost and all that. (Participant 6; Psychiatrist; Facility 3)

In addition, Participant 26 explained:

If a patient is aggressive, it shouldn’t be all about the chemical management… giving medication to calm the patient down. Sometimes, you need to talk to the patient, sometimes, when you get a straitjacket to calm the patient down, can help do the talking. We have seclusion rooms, but our seclusion rooms are not standardized seclusion rooms, where you have hard seclusion rooms… I hear in advanced nations their seclusion rooms are conducive for caring for the patient. Unfortunately, we do not have such seclusion rooms here, and it’s a worry to most of the staff in the ward. (Psychiatrist; Facility 1)

Some MHPs (a clinical psychologist and occupational therapist) supported this view about the lack of equipment and logistics to facilitate comprehensive psychological and vocational services. Moreover, the lack of adequate equipment in managing information for consumers could endanger their privacy and confidentiality, as one MHP described:

If you are supposed to give someone a psychological assessment, it is supposed to be confidential but here, being a large unit we do not have access to computers, and so when you are done with your assessment the write-up has to be done elsewhere, and that can somehow give way to some people who are not supposed to be privy to whatever information you have in that report, and I think that does not also help. (Participant 18; Clinical Psychologist; Facility 2)

Five MHPs further expressed that people with disabilities, such as blindness and physical disabilities, face some barriers in accessing mental health services. These challenges were largely associated with lack of braille for the blind, and an inaccessible physical environment for blind and wheelchair users. For instance, a Registered Mental Health Nurse with 16 years of experience noted, “then for the blind, the braille is a barrier.” In addition, a psychiatrist and a Registered Mental Health Nurse with 4 and 8 years of experience, respectively, stated:

We don’t have a friendly structure for them, things like where a wheelchair should pass and all that because the infrastructure is very old but… if they come in and we think that we need to see them early before other people… because they are disabled and cannot wait in the queue for too long, we do that. (Participant 28; Psychiatrist; Facility 1)

Accessibility is one of our difficult areas because in this ward at least we have 3 blind men, and we feel that their management or treatment should be a little bit different; especially, we have to help them locate their environment but because of maybe the cost involved… (Participant 20; Registered Mental Health Nurse; Facility 2)

### CMO configuration 3: Promoting inclusivity and geographical proximity of services

This case describes that the psychiatric facilities aspire to make services accessible to all consumers by promoting inclusive mental health services. Most MHPs noted that the measures used to promote accessibility and inclusivity were the scheduling of services for clinical days or contacts, prioritizing services for elderly consumers and people with disabilities (e.g., allowing them to skip queues and providing support to meet their needs), and disaggregating in-patient wards based on the categories of consumers and services. Further, the MHPs narrated that they pay for the cost of services for a vagrant or a person with a chronic condition who has no finance or family caregivers. For instance, MHPs from psychiatric facility 3 explained that Mondays are reserved for new cases, Tuesdays for the specialized child and adolescent mental health clinic and the elderly, whereas Wednesdays are for young adults and the middle-aged groups. Some MHPs added that child and adolescent clinics, as well as aged or elderly services, are not incorporated into the general adult population. Lastly, Thursdays are reserved for maintenance clinics or clinical reviews, and Fridays for meetings and academic sessions. A clinical psychologist exemplified the above interpretation as follows:

Our clinic is structured in such a way that Mondays are for new cases; Mondays are days we see only new cases because…so we see new cases on Monday; then, on Tuesdays we have…clinic and the pediatric clinic as well, so we see the elderly and children, and Wednesdays are basically for young adults and the middle age groups, and then Thursdays are maintenance clinic where people that have been seen already come for review and Fridays are when we do a lot of, let’s say, CPDs; so Fridays we do have a lot of meetings, academic sessions, and all of that to at now, we intend to spread it out to have different days and different times for different clinics, but we are overwhelmed and staffing is not so big now, and as we go on we might… (Participant 3; Facility 3)

Similarly, most MHPs from facility 2 mentioned that there are specialist doctors reserved for child and adolescent services. They also narrated that clinical reviews for adult services are scheduled from Mondays to Thursdays, whereas new cases are for Fridays. Although there are schedules for each day for different services, consumers can attend the mental health service at any time, especially in case of emergencies. Lastly, MHPs in facility 1 mentioned that the outpatient department (OPD) clinics are scheduled for different days for different consumer categories. For instance, on Wednesdays, they prioritize children and the elderly, and schedule adult clinics for Monday, Tuesday, and Thursday. However, priority is usually giving to elderly consumers on all these days. MHPs further narrated that the in-patient wards are disaggregated based on the categories of consumers and services. For instance, a female participant with 12 years of experience described this process:

We have a ward for aged, we have a ward for children, and other wards for different conditions we receive. So, it has specifically prepared for such a group. If you are aged, you will not be mixed with the children. You will be separated. (Participant 22; Registered Mental Health Nurse; Facility 1)

The analysis highlights that the scheduling of specialized clinics based on consumer category is aimed at promoting quality and increasing clinical contact, equity, and inclusive services. One MHP explains that scheduling services enables consumers to access a wide range of specialist services, improves contact care, and reduces the waiting time at the psychiatric facilities:

Yes, I think it greatly improved it; in the past, whereas children were seen mixed with the adults, they will queue as long as everybody else, spend the whole day, miss school and things like that, now, because we have a child clinic, which is on Tuesdays and we try to do early by 12:00 pm, we should be done; so even if the child would miss school, they won’t miss the whole day of school, it reduces the waiting time, so it improves the contact care, and we make sure that the services are all concentrated at one place. (Participant 6; Psychiatrist; Facility 3)

Further, most MHPs conveyed that consumers, such as the elderly and people with disabilities are mostly given priority by allowing them to skip queues as well as receive support services. The MHPs noted that they plan the care for consumers based on their individual needs, as echoed by a participant with 6 years of experience:

If a patient comes in with another disability aside the mental illness, there will be [preferential treatment]. The time that the patient will spend at the OPD will reduce, the service providers attend to the patient much more quickly than others, and then, staff will be available to assist that person with disability quicker than others… and if there are other challenges that we would have to deal with… for instance, financial challenges, the facility has a place [social welfare] for such a patient. So it’s easier for such patients with other disabilities to access the services. (Participant 23; Psychiatrist; Facility 1)

### CMO configuration 4: Information, sensitization and awareness encourages mental health quality

This CMO highlights the information sources used to promote quality mental health services among consumers. The majority of MHPs narrated that several platforms, such as psychoeducation sessions, public relation units, outreach programs, and the media (radio and TV as well as social media, such as Facebook, Instagram, and Twitter) were used to create awareness and sensitization about mental health services in communities, schools, and churches. Most MHPs said they provide psychoeducation to consumers and family caregivers (e.g., during outpatient visits, consultation sessions, at the time of discharge, and in the in-patient ward) regarding the importance of medication and the ways to administer such medication as well as manage potential side effects. In particular, some MHPs noted that they explain to consumers and family caregivers about the available medications that could be used to manage any potential side effects from medications. Two MHPs added that in an attempt to provide evidence-based treatment, the pharmacist and practitioners routinely provide an educational session or workshop to enlighten them on new medications. Two participants illustrated this interpretation as follows:

So, during the consultation, after the history and all the examination has been done, we tell the patient about the medication that we are prescribing for them; then, we tell them the reason why they are being given the medication and the side effects. In our pharmacy, they usually have talked to the patient. And sometimes, depending on the medications, they also have information about the medication available to the patient. I know that in certain instances, they give out the leaflet in the medication box to the patients, though not all patients get access to such information. (Participant 23; Psychiatrist; Facility 1)

When they come for the medications, we educate them about the drugs; they educate them on what the drugs can do, if you take them, how it will help treat your condition, the side effects of the drugs, and what to do when you experience some of the side effects of the drugs; there are medications that we use to counter the side effects of the drugs, and we educate them on all of these when they come for their medications at the OPD, and even at the In-Patients, those that have been admitted, so when we educate them and their families about the drugs that are how we do it. (Participant 3; Registered Mental Health Nurse; Facility 3)

Moreover, most MHPs noted that they collaborate with stakeholders (e.g., churches, special educators, physiotherapists, social welfare, faith-based healers, police, NGOs, and schools) in performing specific tasks to create awareness about services, which helps to promote the quality of, and access to, the services as exemplified:

We go to these churches, different churches, to give talks like that to the entire church and in the presence of the pastors as well so that they also get that information. We have also in few instances organized workshops for some of these leaders to come on board with their concerns and the myths that they carry around, and we try to correct those myths and all of that; so it has been a process and it has been ongoing, and as we started some years back, we have seen improvements, we have seen a lot of pastors referring cases, a lot of religious leaders referring cases to the hospital and even calling us personally to tell us that “we have a particular person, and we want that person to come and see you”. (Participant 4; Clinical Psychologist; Facility 3)

Next, the following participants explains:

I do a lot of talks, health talks, psychoeducation at churches, schools and among others, and we also encourage the people who might be suffering from any of the condition… sometimes we give them the triggers/symptoms…. That if you see these triggers, it is good to seek help. (Participant 27; Clinical Psychologist; Facility 1)

With these special schools, I go to these special schools two times in a week either before or after my normal duties at the hospital to check up on the kids and also to check up on the teachers or the therapy providers there, so I visit these centers almost every week. (Participant 2; Occupational Therapist; Facility 3)

MHPs (13/30) highlighted that they embarked on outreach programs and public campaigns to communities and schools to sensitize the public about the available mental health treatment and to help destigmatize mental illness. Four other MHPs mentioned that these advocacy and awareness events are mostly conducted during international days designated for mental health issues, including the World Mental Health Day, the World Suicide Day, the Mental Health Week, and the Mental Health Day. The analysis suggests that MHPs use sensitization and awareness not only to improve public knowledge about services but also to improve the quality of the services.

### CMO configuration 5: Monitoring and evaluating improve mental health service quality

The MHPs presented diverse perspectives regarding approaches used to monitor mental health service quality. The majority narrated that a quality assurance team in the psychiatric facilities conducts quality assessments through internal and external clinical peer reviews, as well as monthly, quarterly, and annual reports and meetings. Most MHPs said that the quality assessment team rely on therapeutic interactions and assessment sheets, or on verbally asking the consumers regarding their activities or observing them. A psychiatrist with 4 years of experience explained that the internal review consists of a team of individuals from various departments, including administration, who come together and use some parameters (e.g., whether MHPs follow the mandated dress code and communicate clearly and politely with consumers; whether consulting rooms meet the basic requirements; and whether medications are prescribed correctly). Some MHPs further noted that after the internal review process, an external peer review team from other psychiatric facilities is invited to review, monitor, and evaluate the progress of the mental health services, using some parameters:

For now, the one available is the peer review mechanism put in place where this facility, for instance, will go and review, monitor, and evaluate the progress made at Pantang [another psychiatric facility] or Ankaful [another psychiatric facility]. Then, Ankaful and Pantang will equally come and monitor our facility. This is done on an annual basis. Before that, the institution itself has a local peer review team that meets to embark on such assignment before the external assignment. (Participant 24; Social Worker; Facility 1)

Some occupational therapists said that there is a weekly clinical review session, as well as assessment sheets to measure the outcome of care. For instance, the clinical review not only sets goals and monitors the consumer’s progress but also identifies any challenges and ways to improve the services:

On our timetable, we have a clinical review every Tuesday afternoon, so whatever we have done for the week, maybe we set goals and targets we want to achieve on our clients; so we meet up and find out how has it been done so far, did we encounter any challenges, what can we do better or should we keep on doing what we are already doing; yes; so every week we have a clinical review. (Participant 11; Occupational Therapist; Facility 2)

There are assessment sheets or forms to do the assessment and measure the outcome, we use that same sheet for the outcome measures and also verbally asking the patient, even how the patient does the therapy in the home, you would want to review and see whether the patient did it right or wrong, and also by observing the patient you know; sometimes by asking the patient to do a simple task, you would want to know whether there is progress or not or even the appearance of the patient can even tell you what is going on, how the patient interacts with other people will tell you what has happened in the course of the therapy. (Participant 2; Occupational Therapist; Facility 3)

The analysis suggested that the internal and external reviews aim to identify the strengths and weakness in the services and to further improve the quality of care provided. Despite these views, some MHPs (7/30) mentioned that the facilities are in the process of obtaining standardized tools to objectively measure the quality of mental health services. For example, a psychiatrist with 4 years of experience explained:

We are coming out with an objective questionnaire this year where we will distribute to some of the patients to assess the quality of service in every way, but what we do presently is to encourage them to let us know if they have any difficulties or if they have any challenges or things they would want us to do and that’s it. (Participant 7; Psychiatrist; Facility 3)

## Discussion

This study used a realistic evaluation methodology to develop a program theory that explores the context and mechanisms that enabled or hindered the quality of mental health services in Ghana. The study has answered the research questions by identifying five contexts and the mechanism that explain the outcome of quality mental health services. The findings have been discussed according to the five CMO configurations:

### CMOc configuration 1: Ripple effects and sustainability of financing

Adequate and reliable sources of financing services are important for consumers, particularly for ensuring secured, sustainable financial protection [20, 26, 27]. However, the current study showed that financing sources of mental health services have a ripple effect, particularly on sustainability as well as the quality of services. Although the government was identified as a mandatory source of financing mental health services, as well as psychiatric medications, the support received from this source was inadequate, leading to over-reliance on internally and externally generated funds. For example, the program theory identified that this challenge translated to limited insurance coverage, out-of-pocket payments, and the high cost of services. In particular, psychiatric facilities were challenged by the infrequent supply of medications, leading to high costs for quality medication. Given this situation, the unintended negative ripple effects were that consumers were unable to afford the cost and to secure quality medications and they purchased alternate lower-cost drugs; further, clinical reviews reduced and noncompliance, as well as adverse side effects, increased.

Compared with the systems in developed countries, mental health systems in LMICs (e.g., Ethiopia, India, Nigeria, Nepal, South Africa, and Uganda.) are confronted with limited reliable and sustainable financial protection, which leads to widespread inequalities in access and high poverty among consumers [20, 23, 26, 27]. In particular, the government funds allocated to mental health services in these settings are inadequate to meet the increasing demand for these services [20, 26, 27]. In some instances, consumers and family caregivers continue to rely on out-of-pocket payments when receiving mental health services. For example, although Ghana has implemented national health insurance for more than a decade, mental health services have not been incorporated into this policy. It is assumed that mental health services should be free by law; however, consumers continue to face this financing burden. As per this program theory, the long-term ripple effect is that consumers could face a relapse, which would subsequently affect the clinical and personal recovery journey [28]. In this scenario, the opportunities that exist could be the integration of mental health services into ongoing reforms to national insurance schemes. Another avenue to overcome the financial burden is the integration of mental health into primary care services, as recommended by the WHO. The inclusion of mental health services could provide adequate sustainable financial protection for consumers.

### CMOc configuration 3: Unavailability of modern equipment and logistics to support holistic services

Accessibility to modern equipment and to logistics constitutes one of the ways to promote access to quality mental health services [20, 29, 30]. Such equipment and logistics could help MHPs to provide quality services to consumers. The findings suggested that even though the psychiatric facilities were expected to operate a holistic/integrated philosophy of care, they lacked the relevant equipment and logistics (e.g., ECT machines, straitjackets, and psychological and vocational equipment). As per this program theory, such challenges compromise the quality of mental health services and thus increase the average working hours spent on consumers, potential injuries, inconvenience, and time taken for clinical and personal recovery, as well as endanger the privacy and confidentiality. The limited modern equipment and logistics could be attributed to several factors, including the low priority of mental health services as well as weak mental health systems. The mental health systems in Ghana have historically faced the challenge of the weak supply of equipment and logistics [20, 29]. This situation is consistent with the finding of previous studies that the health systems in most LMICs have limited priority for mental illness, which subsequently leads to limited policy, legislation, and regulation to back the supply of modern equipment and logistics that could enhance service delivery [31–33].

Thus, these equipment- and logistics-related challenges could have ripple effects on the quality improvement of services. For example, Kilbourne, Keyser [2] reported that several barriers affect the implementation of quality assurance indicators, including equipment and logistics. These challenges could endanger the quality of mental health services that consumers and MHPs receive. As per the program theory, government stakeholders are encouraged to provide adequate modern equipment and logistics to psychiatric facilities in the quest to promote quality mental health services. For example, psychiatric facilities should be modernized with the current equipment and logistics needed to provide evidence-based treatment to consumers.

### CMOc configuration 3: Promoting inclusivity and geographical proximity of services

Several efforts have been launched globally to promote an inclusive mental health service that is friendly to all consumers. Such services could improve consumers’ access to holistic services and further promote their recovery journey [6, 28]. The study indicated that the psychiatric facilities embarked on several initiatives in the quest to promote inclusivity and address geographical proximity, which include scheduling of services for clinical days or contacts, prioritizing services for elderly consumers and people with disabilities (e.g., allowing them to skip queues and providing support to meet their needs), disaggregating in-patient wards by consumer category, and paying for the cost of services for vulnerable consumers. The program theory suggested that these mechanisms could promote the quality of mental health, such as by increasing clinical contact and equity, providing access to a wide range of specialist services, reducing the waiting time at the psychiatric facilities, and enhancing inclusive services. The findings suggest that incorporating these mechanisms into the routine clinical and quality assurance practices could not only enhance inclusivity and quality but also promote access to services among the elderly and people with disabilities, who are perceived as vulnerable. Consistent with a previous study in the US setting, such practices are relevant to make mental health services responsive. Given these findings, we recommend that mental health policies should continuously integrate the needs of these populations into service planning and delivery.

### CMO configuration 4: Information, sensitization, and awareness encourage mental health quality

The use of effective communication and information sources to make services known to consumers and family caregivers constitutes one of the strategies to promote the quality of services [4, 20]. The health literacy of consumers and family caregivers is also relevant when promoting mental health and education. The study showed that psychiatric facilities used several mediums, such as psychoeducation, news media, and outreach programs to promote and create awareness about mental health services. As per the program theory, psychiatric facilities used such platforms to provide psychoeducation about medications and side effects and to enlighten MHPs on new medications. Similarly, these mechanisms were used to promote evidence-based treatment, for example, to enlighten providers on new medications, manage potential side effects, improve the public knowledge about available mental health treatments, and reduce stigmatizing attitudes.

In addition, these mechanisms were expected to enhance the quality of mental health services. For example, previous studies have concluded that stigmatizing attitudes and acceptance of faith-healing in Ghana are increasing [21, 34]; therefore, promoting access to information as well as awareness about the available mental health services could increase consumers’ access to services and further address the issues related to geographical proximity. Using existing media platforms to create awareness about services should be promoted and encouraged among psychiatric facilities. As in some developed countries, such as Australia [35, 36], innovative approaches, such as telemedicine services, could be introduced where consumers can use video conferencing equipment and internet services to book an appointment and to consult MHPs. Such measures could provide consumers with knowledge about their condition through online or text messaging as well as video conferencing. For example, Saurman, Perkins [36] and Lessing and Blignault [35] concluded that telehealth can increase the access to mental health services of people in rural and remote areas in Australia.

### CMOc configuration 5: Monitoring and evaluating improve mental health service quality

Measuring the quality of mental health services using relevant indicators is an important step toward monitoring quality improvement [37]. In particular, standardized measures integrated into clinical practices to monitor and track outcomes of services over time could form the foundation of performance improvement [2, 37]. The current findings suggest that several quality assessment measures, such as internal and external clinical peer review and monthly, quarterly, and annual reporting are used to evaluate the clinical outcomes of mental health services. Specifically, allied health professionals used therapeutic interactions, such as verbal communication and observation of consumers’ activities to monitor and evaluate the clinical outcomes of services. As per the program theory, these mechanisms of quality assurance not only help to monitor and evaluate the progress of the mental health services but helps MHPs to identify challenges and set targets on how to improve the services.

Although these mechanisms are relevant in monitoring the progress of services, there is little emphasis on the mental health system report, particularly on the recovery or symptomatology of consumers. In this regard, psychiatric facilities have recently implemented a Mental Health Information System (MHIS) to document consumer admissions, diagnoses, discharge date, and medication prescribed [27, 38, 39], but place limited emphasis on the systematic measure of clinical recoveries that could inform decisions. Hence, mental health services are unable to document consistent information regarding clinical recovery services. Unlike in developed countries [2], the mental health service system in Ghana is at the developing or transformation stage, and therefore faces a challenge with the health information system. The MHIS was expected to improve the effectiveness of services, thus enabling managers and service providers to make more informed decisions for improving the quality. Nevertheless, the current findings confirm those of previous studies, which have concluded that information management systems for mental health services are poor, particularly in developing countries [39–41]. For example, Kpobi, Swartz [41] identified that the optimal use of the current MHIS is faced with several implementation challenges, which include increased workloads, inadequate clinical skills and knowledge of staff, as well as the absence of logistic support to operate and maintain the system.

Given these findings, stakeholders from government agencies and psychiatric facilities are encouraged to use new approaches and technologies to capture adequate mental health data that could inform clinical practices. For example, sensor data and machine learning could be incorporated into the existing MHIS to allow automatic and continuous monitoring of mental conditions. Thus, psychiatric facilities could move from mere administrative data collection to its analysis and dissemination, which would be used to inform clinical practice.

### Limitations

Several limitations need to be acknowledged. First, this component of this article is limited to the response from purposively selected MHPs in three psychiatric facilities without the perspectives of mental health policy planners from government ministries as well as consumers and family caregivers. The selection of the three psychiatric facilities could lead to potential bias. Despite this, the researchers have enhanced the trustworthiness of data collection and documentation according to the seven criteria of Pawson and Tilley [16], which are transparency, accuracy, purposivity, utility, propriety, accessibility, and specificity. The methodology informing this CMO configuration was collaboratively developed by experts in research methodology. For example, the research team first developed, confirmed, and discussed the methodology before implementing it. Other methodological considerations governing this study have been published elsewhere. Moreover, the thematic analysis process was subjected to coding by consensus, member checking, and a series of debriefing sessions. The findings have been compared with those of the international literature on quality mental health services.

## Conclusion

In this study, we used a realistic evaluation method to explore service providers’ insights about contextual factors and mechanisms that influence the outcome of mental health services. We identified five CMO configurations. Specifically, we showed that there were ripple effects and limited sustainable financial protection for consumers, which subsequently influence the mental health service quality. The study also concludes that even though the psychiatric facilities were expected to operate a holistic/integrated philosophy of care, they lack the relevant modern equipment and logistics that could facilitate quality mental health services. Despite these challenges, the program theory identified several mechanisms used to promote the inclusivity of vulnerable consumers as well as close the gap of geographical proximity. In addition, the program theory recommended several mechanisms used to advocate, and create awareness about mental health services and further promote the quality of services. In addition, the program theory has suggested that the monitoring and the evaluation of mental health services could be strengthened through effective use of new technologies and innovations.

### Implication and recommendation

We answered the research questions by identifying the contextual factors and mechanisms that could promote quality mental health services. The findings from the study are relevant to inform policy, advocacy, mental health nursing practices, and the education of MHPs and students. Next, we make several recommendations for policy and mental health practices based on the program theory. Given the ripple effect of financial risk protection, the study recommends that stakeholders should facilitate efforts toward the integration of mental health services into ongoing reforms to national insurance schemes. The inclusion of mental health services could provide adequate sustainable financial protection for consumers. Government stakeholders are also encouraged to provide adequate modern equipment and logistics to psychiatric facilities in the quest to promote quality mental health services. For example, psychiatric facilities should be modernized with the current equipment and logistics needed to provide evidence-based treatment for consumers. As per the program theory, the study recommends that current mechanisms used to promote inclusive services for people with disabilities, the elderly, and children should be prioritized and monitored in the quality assurance of mental health services. For example, the priority given to this population should be integrated into policy documents and legislation and be periodically reviewed to ensure the continuous participation and the inclusion of this population. This approach could help address the inequalities in access to services among this population.

Moreover, mechanisms and platforms used to advocate and promote mental health services should also be prioritized and applied effectively for continued increase in public participation and the use of biomedical treatment for mental health services. For example, government stakeholders should adhere to the mechanisms identified from this program theory to inform public awareness campaigns. Given the weakness in monitoring and evaluating services from the existing MHIS, based on the program theory we recommend incorporating new approaches and technologies to capture adequate mental health data that could inform clinical decisions and practices. For instance, sensor data and machine learning could be incorporated into the existing MHIS to allow automatic and continuous monitoring of mental conditions. We recommend that the current context, mechanism, and outcomes of this program theory should be integrated into training courses for MHPs to update their knowledge and skills. Recognising that the present study did not consider mental health policy planners from government ministries as well as consumers and family caregivers, future research should use a participatory approach to integrate the perspectives of all stakeholders. This could help to plan and develop quality mental health services that improves the overall wellbeing of consumers.

## Supplementary Information

Supplementary Material 1.

## Data Availability

The datasets used and/or analysed during the current study are available from the corresponding author on reasonable request. All data collection tools, including the interview guide, have been uploaded as supplementary files.

## Abbreviations

CMHN: Community Mental Health Nursing
CMHO: Community Mental Health Office
CMO: Context + Mechanism = Outcome
CPDs: Continuing Professional Development
DFID: Department for International Development
ECT: Electroconvulsive therapy
HREC: Human Research Ethics Committee
LMICs: Low Middle-Income Countries
MHIS: Mental Health Information System
MHPs: Mental Health Professionals
NHIS: National Health Insurance Scheme
OPD: Outpatient Department
RMN: Registered Mental Health Nursing
