# Supplementary material for: Providers’ insight into quality mental health services – Context-Mechanism-Outcome (CMO) approach
Source: BMC Health Serv Res. 2025 Feb 17;25:264. doi: 10.1186/s12913-025-12372-x (PMC11834691; doi:10.1186/s12913-025-12372-x)
Supplement: Supplementary file 1 — Supplementary Material 1. [file 12913_2025_12372_MOESM1_ESM.doc]

**Interview guide – Mental Health Professionals**

Authors address (XXX)

**Interview guide for the Research Project:**

**The quality of mental health services in Ghana: Providers and Consumers perspective**

*Dear Sir/Madam,*

You are invited to participate in the research project identified above, which is being conducted by PhD student XXXX is enrolled at University of XXX. XXX is supervised by XXX and XXX.

*The attached interview guide asks you to respond to questions concerning your experiences with the quality of mental health services you provide to consumers. The interview is part of my doctoral studies being supervised by the XXX at the University of XXX. It is appreciated if you could participate in this interview. Your opinions are highly valued and essential to improve the kind of mental health service in Ghana****.*** *Your response will be recorded using an audio tape recorder, however, your name or any other personal information will NOT be used in the research study to maintain your confidentiality and anonymity. No individual will be identified accidentally. No individual details will be disclosed to anyone. Your interview will be transcribed using a false name (pseudonym). Code numbers and pseudonyms will be used in place of names throughout the research process.*

***Questions***

1. **Tell me about the availability of mental health services to consumers?**

Probe

1. What is the range of mental health services? clues – *psychiatric medication, psychoeducation, psychosocial services, mindfulness mindfulness-based interventions (conflict management, coping skills), physical health education, cognitive behaviour and family therapy as well as leisure and recreational activities?*
2. How do you ensure the use of modern equipment and logistics to facilitate comprehensive assessments?
3. How does this contribute to quality mental health services?
4. **Tell me about the process involved in the supply and prescription of medications to consumers?**

Probe

1. From where do you get medications at this psychiatric unit?
2. What is the timely availability of such medications?
3. How do you prescribe medications to consumers? *Clues -Choice of medication is based on individual factors (desirability of sedation, previous response to a drug treatment including adverse reactions) co-morbid psychiatric or medical conditions?*
4. *How do you ensure the use of evidence-based and local protocols to prescribe medications?*
5. How do you ensure that information on medication is accessible to the consumer? *Clues - clear and accessible information provided with the prescribed psychiatric drug including information about potential benefits and unwanted side effects? Provision of clear and accessible information to consumers about drugs?*
6. **Tell me about the technical competency of mental health professionals in this facility?**

Probe

1. What is the technical competency of providers? Clues –clinicians’ knowledge about appropriate intervention, ability to use best practices and to accurately assess consumer problems?
2. How does this work in practice?
3. How does this contribute to the quality of mental health services?
4. **Tell me about the training and professional development plan and programmes available for you?**

Probe

1. Have you attended any in-service training this year?
2. How often do you attend the in-service training? In which areas do you receive in-service training?
3. What are the training needs?
4. How does this contribute to the quality of mental health services?
5. **Tell me about the extent of access to mental health services to consumers?**

Probe

1. How do you ensure equity of access regardless of gender, disability status, age group and geographical location? Clues – considering the needs of people with language issues, information for consumers and family caregivers, medical records?
2. How does this contribute to the quality of mental health services?
3. **Tell me about the extent at which consumers are involved in the treatment plans?**

Probe

1. How do you involve consumers in the formulation and delivery of their care? *Clue – the treatment plans are individually tailored for each consumer? Consumers are informed, where appropriate, about the reasons for referral to a specialist or other professionals? listening to consumers and taking them seriously? Mental health professionals are aware of the potential impact of a mental health problem on consumers’ behaviour? Mental health professionals are aware of stigmatizing attitudes against consumers?*
2. How do you handle the therapeutic relationship with consumers? *Clues Treating consumers with respect, courtesy and considerations irrespective of age, gender, religion and cultural identity?*
3. How does this contribute to quality mental health services?
4. **Tell me about your experiences working with consumers of mental health services?**

Probe

1. How does their socio-demographic background facilitate the quality of mental health services? Clues – the c*linical history, gender, cultural issues?*
2. How does your therapeutic relationship with consumers influence the quality of mental health services?
3. **Tell me about the approaches and activities used to create awareness about mental health services?**

Probe

1. *How do you create awareness and advocate for mental health treatment?*
2. *How do you collaborate with other stakeholders?*
3. *How does it work in practice?*
4. *How does this contribute to the quality of mental health services?*
5. **Tell me about the approaches you use to monitor the quality of mental health services in this psychiatric unit?**

Probe

1. *How often do you assess the quality of the services?*
2. *How does this work in practice? Clues – the written protocols and mechanisms in place for monitoring prescribing psychiatric drugs? Any mechanisms to address the side effects of medications?*
3. *How does this improve the quality of mental health services?*
4. **Tell me about the sources of financing mental health services?**

Probe

1. How do the sources of financing mental health services contribute to the quality of services?
2. **Tell me about your understanding of recovery?**

*Probe*

1. What comes to your mind when we mention of recovery in mental health? *Clues – the ability of consumers to achieving inner strength and willpower, changes in consumers lifestyle and reclaiming various aspects of self? Achieving a sense of personal hopes and optimism about the future, a growing sense of agency and autonomy? Consumers goal and priorities? Are consumers having a sense of self-determination, self-understanding, self-acceptance, self-control and personal transformation? Consumers optimum level of functioning, improvement of symptoms, returning to a healthy state, or pre-illness state, achieving a normative life and connectedness?*
2. Who educated you about recovery in mental health?
3. How has your understanding help in providing services to consumers?
4. **What is the existing recovery plan for consumers receiving mental health services?**

**Probe**

1. How does the psychiatric facility achieve recovery in mental health? Clues – recovery through mental health systems report, personal recovery process ie setting personal goals, discharging to residential facilities?
2. How does the process work in practice?
3. **What is your expectation towards the personal recovery goals of consumers when discharged from this facility?**

Probe

1. What do you expect consumers to do when discharged from this facility? Clues – go back to work, go back to school, and participate in community and church activities? Taking decision on my own? How does that work in practice?
2. How can you help consumers to achieve this personal recovery goal?
3. How can the personal recovery goals of consumers be achieved?
